# Development and Validation of a New Method to Measure Walking Speed in Free-Living Environments Using the Actibelt® Platform

**DOI:** 10.1371/journal.pone.0023080

**Published:** 2011-08-05

**Authors:** Michaela Schimpl, Christian Lederer, Martin Daumer

**Affiliations:** 1 Trium Analysis Online GmbH, Munich, Germany; 2 Sylvia Lawry Centre for Multiple Sclerosis Research e.V. - The Human Motion Institute, Munich, Germany; Institute Biomedical Research August Pi Sunyer (IDIBAPS) - Hospital Clinic of Barcelona, Spain

## Abstract

Walking speed is a fundamental indicator for human well-being. In a clinical setting, walking speed is typically measured by means of walking tests using different protocols. However, walking speed obtained in this way is unlikely to be representative of the conditions in a free-living environment. Recently, mobile accelerometry has opened up the possibility to extract walking speed from long-time observations in free-living individuals, but the validity of these measurements needs to be determined. In this investigation, we have developed algorithms for walking speed prediction based on 3D accelerometry data (actibelt®) and created a framework using a standardized data set with gold standard annotations to facilitate the validation and comparison of these algorithms. For this purpose 17 healthy subjects operated a newly developed mobile gold standard while walking/running on an indoor track. Subsequently, the validity of 12 candidate algorithms for walking speed prediction ranging from well-known simple approaches like combining step length with frequency to more sophisticated algorithms such as linear and non-linear models was assessed using statistical measures. As a result, a novel algorithm employing support vector regression was found to perform best with a concordance correlation coefficient of 0.93 (95%CI 0.92–0.94) and a coverage probability CP1 of 0.46 (95%CI 0.12–0.70) for a deviation of 0.1 m/s (CP2 0.78, CP3 0.94) when compared to the mobile gold standard while walking indoors. A smaller outdoor experiment confirmed those results with even better coverage probability. We conclude that walking speed thus obtained has the potential to help establish walking speed in free-living environments as a patient-oriented outcome measure.

## Introduction

Walking speed is increasingly considered to be an important indicator of a subject's health status [1]. Previously, changes in walking speed measured in a clinical setting have been accepted by the FDA as primary outcome in a phase III clinical trial in multiple sclerosis (MS) [2]. Only recently, the EMA has reconsidered its previous negative opinion towards fampridine – a drug intended to improve walking ability in MS patients – provided that long-term efficacy and clinically meaningful outcomes for walking ability will be investigated by the sponsor [3].

In a clinical setting, walking speed is typically measured by either short or long walking tests which have been demonstrated to be a powerful predictor for survival, disability, hospitalization, dementia and falls [4], [5]. However, the 10-meter walk test, a typical example for short walking tests, shows a considerable amount of measurement noise and bias due to its brevity [6] and variations of measurement protocols. Long walking tests such as the 6-minute walking test or 500-meter walking test may often not be feasible to embed into daily clinical routine because of space [7] and time requirements as well as logistic efforts, can vary with patients' motivation and learning effect [8] and even if performed in a highly standardized setting show a high day-to-day variability [9], [10]. Moreover, discrepancies between objective measurements and patient-reported limitations of walking ability are often not reflected by these tests [11] with self-report instruments such as physical activity questionnaires lacking content and construct validity as well as reliability [12]. Consequently, there is a need for methods to determine walking speed as a key descriptor and predictor of physical activity which have less bias, higher precision and better practicability. Therefore walking speed should ideally be measurable also in uncontrolled environments, since longer tests can better capture an individual's walking performance [13]. However, preparatory work using appropriate gold standards prior to application in a free-living environment is necessary to determine the accuracy of such a new method under conditions which resemble those in a free-living environment.

GPS devices are inappropriate for usage in free-living conditions as they are power-consuming, restricted to outdoor usage, dependent on weather conditions and satellite availability [14] and entail potentially problematic data privacy issues. Instrumented walkways (e.g. GAITRite® [15]) have a high accuracy but are confined to a laboratory setting. Other available mobile devices to measure walking speed in free-living environments such as Pedar [16] and IDEEA [17] have restricted usability with a standard operating time of less than seven days.

Mobile accelerometry can overcome these limitations, but type and accuracy of measured parameters strongly depend on the type of accelerometer. Currently there are several mobile accelerometry devices on the market claiming to be able to assess walking speed, but in order to yield usable high-quality data, user-friendliness is of utmost importance. Systems consisting of five accelerometers attached to chest, thighs and forefoot [18], three accelerometers mounted on waist and thighs [19] or a single accelerometer integrated in a tight-fitting elastic short [20] are unlikely to gain high user acceptance for long-term monitoring due to their cumbersomeness resulting in incomplete data sets. Other accelerometers which are attached to the ankle [21] may cause discomfort to the user due to their resemblance to electronic tags in addition to being only able to capture the motion of one leg similar to the thigh-worn activPAL [22]. Comparable devices such as GENEA [23] and Philips DirectLife [24] lack a standardized method of attachment. In general, this can lead to a sub-optimal performance of algorithms [25].

The actibelt® platform contains a tri-axial accelerometer integrated in a belt buckle which the wearer fixes around the waist by either a leather or elasticated belt [26]. This design was chosen for two reasons: First, the unobtrusiveness of this set-up ensures a high user acceptance which has been demonstrated in an independent assessment [27]; secondly, from a biomechanical point of view the location where a belt buckle is usually placed is ideal because it is close to the body's centre of mass and the axis of symmetry in the sagittal plane which allows to capture asymmetries of both lower extremities.

Although treadmills are widely-used tools in the context of research related to gait, exercise and sports due to their convenience, it is known that treadmill walking significantly differs from overground walking in terms of kinetics and kinematics [28], [29] and therefore has unclear ecological validity. For this reason a new mobile gold standard was developed to measure the walking speed of a freely-moving individual.

This investigation describes the development and validation of various algorithms for walking speed prediction using actibelt technology suitable for long-term monitoring with an additional quality assessment framework including a newly developed mobile gold standard which allows a direct comparison of algorithms based on a controlled data set and appropriate statistical measures.

## Methods

### actibelt®

The actibelt® is a tri-axial accelerometer (512 MB memory corresponding to 10 days of continuous recording, sampling frequency 100 Hz, battery life 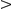 20 days) placed inside a belt buckle [26]. The design is unobtrusive and allows the device to be closely located to the subject's centre of mass. It can either be used for long-term monitoring in a free-living environment (“week-in-a-box”) or activity assessment in a clinical setting (“rapid tests”).

### Mobile gold standard

**Figure 1:**
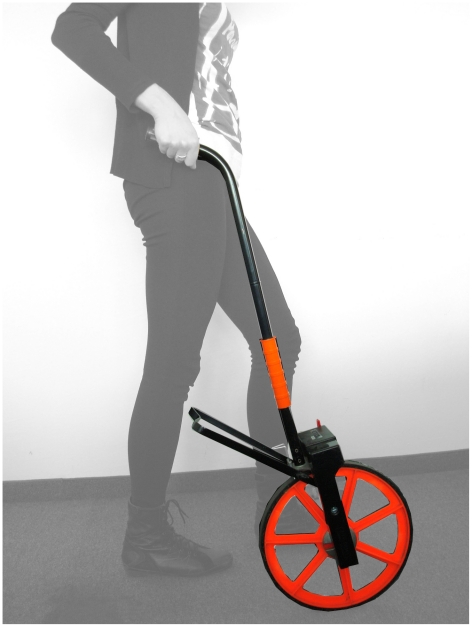
Mobile gold standard. Example of a test subject operating the mobile gold standard.

The mobile gold standard consists of a high-end bicycle computer (CS600X, Polar Electro, Kempele, Finland) mounted on a perambulator (M10, Geofennel, Baunatal, Germany) (Figure 1).

A perambulator is a device commonly used for land surveying to measure distance covered with a calibrated mechanism to record distance for each revolution of the wheel by pushing the wheel overground. The device used in this study has a wheel circumference of 1 m and a high-precision counter accurate to within a centimetre. The speedometer is usually mounted on a bicycle to record speed, heart rate and altitude of an athlete. For this study the bicycle computer was mounted on the wheel of the perambulator to measure walking speed of a subject operating the perambulator. The speed measurement is wirelessly transmitted to a small hand-held computer from which data can then be exported to text files using provided software (ProTrainer 5, Polar Electro, Kempele, Finland) for further analysis.

With this mobile gold standard speed can be measured in the range of 0 to 127 km/h with a frequency of 1 Hz. Agreement between velocities measured by the mobile gold standard and treadmill is very high with a concordance correlation coefficient of 0.99 for speeds ranging from 1 to 8 km/h.

Besides overcoming the limitations of the treadmill, an advantage of this set-up is the tight coupling of the perambulator with the bicycle computer which allows to correct for potential aberrations of the bicycle computer by scaling the obtained values with an individually calculated factor based on the distance measured by the perambulator.

### Speed prediction algorithms

Most algorithms for walking speed prediction use one of following basic principles: combining step length with step frequency, linear regression (LR), support vector regression (SVR) or integration. Different variations and models for each algorithm subgroup were evaluated.

#### Combining step length with step frequency. Step length (default)

The individual's walking step length is multiplied by the number of steps to obtain distance. Subsequently, walking speed can be deduced taking into account the time needed to cover this distance.

#### Step length (walk/run)

The above described approach is extended by introducing a second step length which is applied during running.

#### Step length (pendulum)

Assuming an inverted pendulum model for human walking, the above described approach is extended by estimating step length from the upward and downward acceleration using a geometrical formula taking a subject's leg length into account [30].

#### Walk ratio (default)

This approach exploits the fact that the ratio between step length (measured in meters) and step frequency (measured in steps per minute) – the so-called walk ratio – is found to be relatively constant across a range of different walking speeds [31]. Assuming that the walk ratio is a universally applicable parameter and step frequency can be extracted from the actibelt® signal, the step length can be determined.

#### Walk ratio (calibrated)

The above described approach is extended by not assuming a universally valid walk ratio for all individuals but rather calibrating the walk ratio based on an individual's step length and frequency.

#### Linear and support vector regression

Table 1 shows the computation and application for all features based on step intervals used in the models for linear and support vector regression.

**Table 1:** Step features.

Feature	Computation	MA-model	C-model	Energy-model	RBWE-model
Minimum	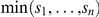				
Maximum	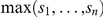		✓ (y)		
Mean	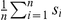		✓ (z, e)	✓ (e)	
Sum	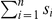		✓ (x, z)		
Sum of absolute values	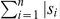		✓ (y, z)	✓ (x, y, z)	✓ (z)
Range	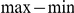				
Step duration	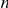		✓		✓
Root mean squared (RMS)	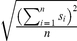		✓ (x, z, e)		✓ (z)
Maximum-mean	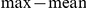	(x)	✓ (x)		✓ (x)
Step amplitude	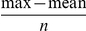		✓ (x)		✓ (x)
Min/max amplitude duration					

Characteristics calculated for each detected step 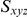 consisting of 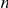 values 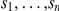. Each of the features listed above (except for the step duration) was computed for each axis 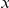, 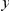, 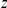 as well as for the energy 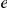 derived from the actibelt® signal which results in a total of 41 features. The columns three to six indicate which features and axes were used for the respective models.

For each detected step in the actibelt® signal, each feature was calculated seperately for the vertical, antero-posterior and medio-lateral (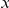, 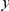 and 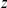, respectively) acceleration as well as for the so-called energy 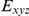 consisting of 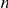 values 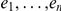 (Equation 1) of a tri-axial acceleration signal resulting in a total of 41 features.
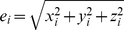
(1)


Except for the energy-model, all features were found via automated backward feature selection. The feature set for the energy-model was determined by the fact that the sum of absolute values of an accelerometer has been found to contribute significantly to models which were used to estimate energy expenditure [32]. For this reason, the four extracted features are the sum of absolute values for each axis as well as the mean energy of the acceleration signal (Table 1).

Features for training the linear and support vector regression were taken from an independent data set of the institute's accelerometry warehouse. A total of 15 subjects (50% male, aged 40 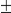 24 years) participated in three different outdoor protocols with speeds ranging from slow walking over jogging to sprinting. Out of these 15 subjects, 8 participants also took part in the below mentioned indoor and outdoor ecological validity experiments. All gait speeds were self-selected and subjects wore their usual every-day shoes. Since this data set was collected before the mobile gold standard had been available, walking speed for each participant and stretch of way was calculated as the ratio between distance covered and time needed to cover this distance.

All SVR models were fitted using a radial kernel. Characteristic parameters for the SVR models were found through 10-fold crossvalidation using a grid search over a supplied range of values (Table 2).

**Table 2:** SVR models.

	MA-model	C-model	Energy-model
C	100	5	64
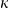	0.03	0.0024	0.004
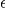	0.2	0.01	0.00049
MSE	0.092	0.063	0.065

Parameters for the various SVR models which were found through 10-fold cross-validation using a grid search over a supplied range of values and total mean squared error (MSE) during cross-validation.

#### Integration

Since acceleration 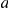 is defined as the rate of change of velocity 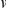, 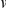 can can be directly obtained by integrating acceleration between two points in time 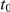 and 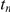 after adjusting for integration drift. As a pre-processing step, a fourth-order, low-pass Butterworth filter with a cut-off frequency of 0.1 Hz was applied [30].

### Pre-processing

Since the actibelt® and the mobile gold standard both measure with different frequencies (100 Hz and 1 Hz, respectively), it is necessary to execute several pre-processing steps in order to match the two signals. To simplify this process the data acquisition protocol was designed to incorporate inactivity periods of 15 seconds between different speed levels. Subsequently, both signals can be scanned for the respective regions of interest and synchronized to within a second.

In order to avoid boundary effects, which can occur when the subject starts or stops walking/running due to the fact that it may take several seconds for the subject to resume a regular rhythm when accelerating or decelerating, only the middle portion of each interval was used for the subsequent data analysis, that is the first and last five seconds of each interval were cut off.

Step intervals which were later on used by each algorithm were automatically detected by a separately validated step counting algorithm. This algorithm uses a simple peak detection method with different delays (before detecting a new step) and thresholds for walking and running.

### Participants and protocols

In order to assess the validity of our method to measure walking speed in free-living environments, we aimed to create situations which best resemble those found under free-living conditions. Since people tend to spend only 2–3 hours per day outdoors on average [33] (which is roughly about 10–15% of their 16 waking hours), the major part of our experiment was for testing indoor ecological validity. In order to further confirm the ecological validity outdoors, a separate experiment was performed with a subset of participants.

#### Indoor ecological validity

A total of 17 healthy subjects (41% male, aged 32 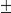 15 years) were recruited from the staff and student population of the Sylvia Lawry Centre for Multiple Sclerosis Research in Munich. Subjects were instructed to walk at five different self-selected gait speeds ranging from slow walking to running (Table 3) for 1.5 minutes each on a 79.0 m (35.0 m 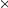 4.5 m) long and 1.6 m wide circular indoor track while operating the above described mobile gold standard and wearing an actibelt® device attached to the waist. Change of gait speed was triggered by an automatic beep of the mobile gold standard followed by a break of 15 seconds. Additionally, each subject was accompanied by an investigator who gave additional instructions when to start and stop, reset the counter of the measurement wheel after each speed level and took down notes regarding the respective experiment. All subjects gave written informed consent. The study was approved by the SLCMSR Local Ethics and Validation Committee (REVA 0001/20100112).

**Table 3:** Speed levels.

Speed level	Mean speed (sd)
Slow walking	0.65 (0.21)
Normal walking	1.11 (0.17)
Fast walking	1.47 (0.20)
Even faster walking	1.83 (0.25)
Running	2.70 (0.46)

Mean gait speed in m/s and standard deviation (sd) for each speed level as measured by the mobile gold standard.

#### Outdoor ecological validity

A subset of subjects (n = 2, 50% male, aged 35 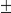 16 years) also participated in an additional experiment with the aim to approximate another important aspect of real-life situations. For this purpose the subjects were instructed to walk outdoors on a 812 m long pavement with increasing walking speed ranging from slow walking to very fast walking peaking approximately after having covered half of the total distance followed by a gradual decrease of walking speed until the end of the way was reached. Walking speed was self-selected without any predefined speed or time intervals.

### Statistical analysis

The use of Pearson's correlation coefficient 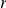 was deliberately avoided as it is an inappropriate measure for assessing agreement between two methods [34]. Instead limits of agreement (LOA), coverage probability (CP) and concordance correlation coefficient (CCC) were used to assess agreement as a joint parameter for accuracy and precision [35] between walking speed measured by the mobile gold standard and estimated values.

Bland-Altman's LOA aid in judging the degree of variation of agreement, whether the measurements are affected by a systematic error and whether the differences between methods are dependent upon the mean [34]. If discrepancies between the two methods as high as the limits of agreement are not of clinical importance, the two methods can be used interchangeably.

CP represents the probability that the difference 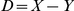 between two measurements 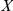 and 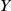 is within a predefined boundary 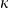 [35]. Since CP is a probability, it is scaled to be between 0 and 1. Usually 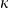 is set to the maximum allowed difference between two methods. If a large proportion of the data lies within the 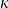 boundary and meets a predefined sufficient coverage probability level, the two methods can be used interchangeably.

The CCC is a modified correlation coefficient which – unlike the well-known Pearson correlation coefficient 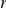 – is able to capture any deviation from the line of equality [36].

All calculations were done using R 2.10.1 [37] with the “e1071” package for SVR support [38] based on the library “libsvm” [39].

## Results and Discussion

**Figure 2:**
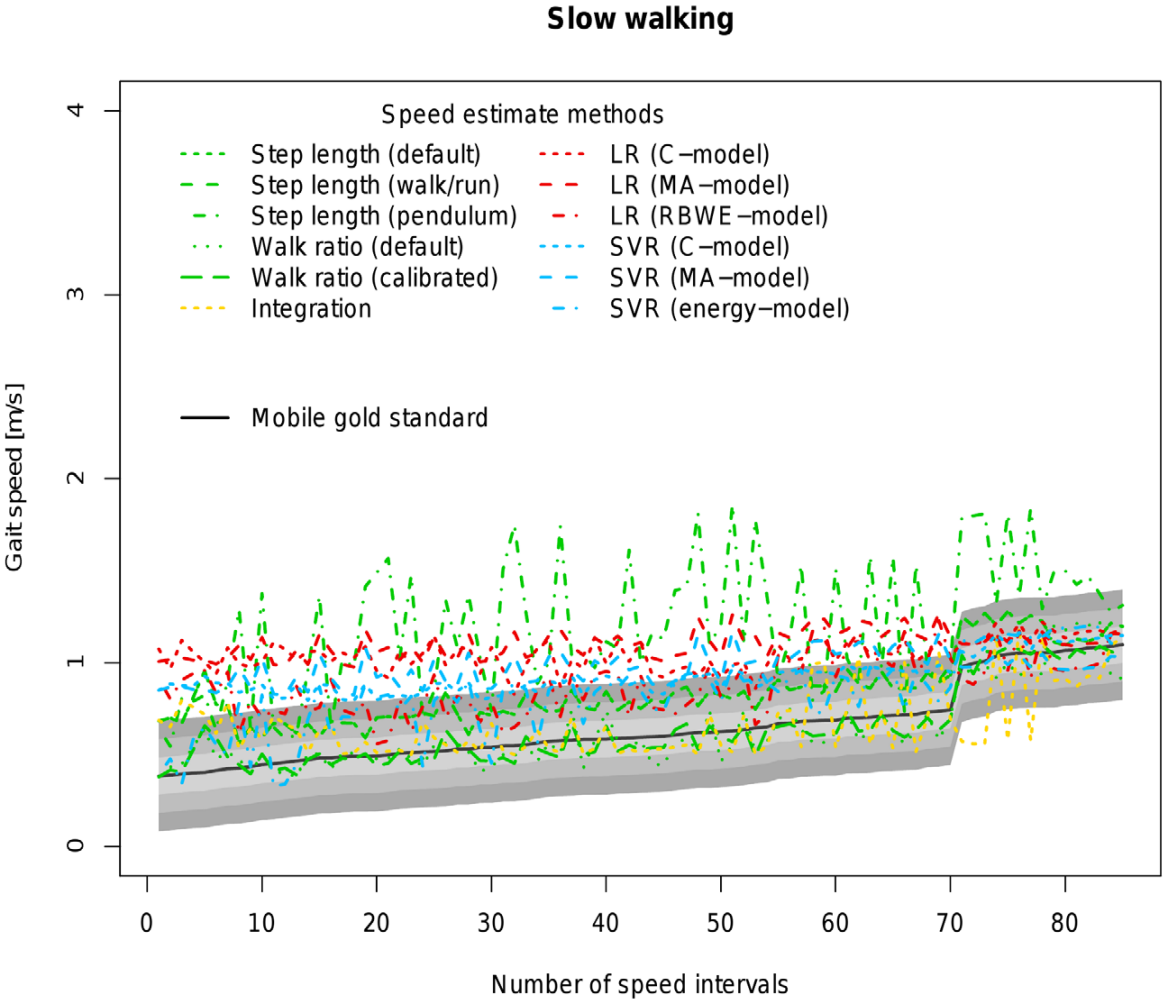
Visualization of coverage probability for slow walking in the indoor ecological validation. The black solid line represents speed as measured by the mobile gold standard for slow walking. Green, yellow, red and blue lines in different linestyles represent different speed estimates by different algorithms and models. The filled areas colored from light to dark grey around the black solid line indicate coverage probality levels from 0.1 to 0.3 m/s. Speed intervals are sorted increasingly across all participants for reasons of clarity and readability.

**Figure 3:**
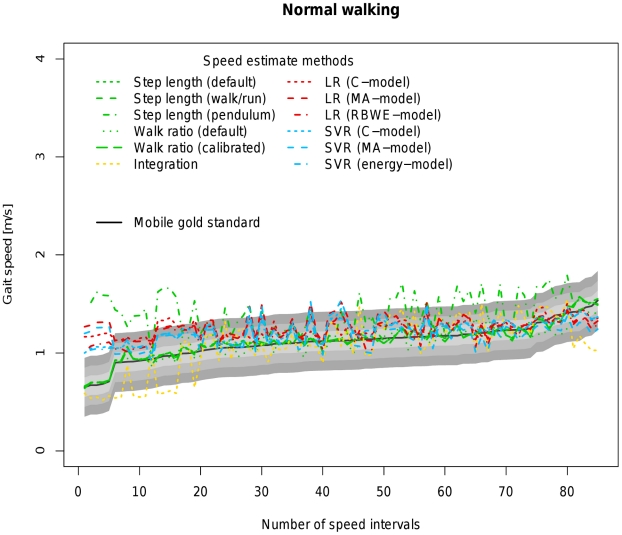
Visualization of coverage probability for normal walking in the indoor ecological validation. The black solid line represents speed as measured by the mobile gold standard for normal walking. Green, yellow, red and blue lines in different linestyles represent different speed estimates by different algorithms and models. The filled areas colored from light to dark grey around the black solid line indicate coverage probality levels from 0.1 to 0.3 m/s. Speed intervals are sorted increasingly across all participants for reasons of clarity and readability.

**Figure 4:**
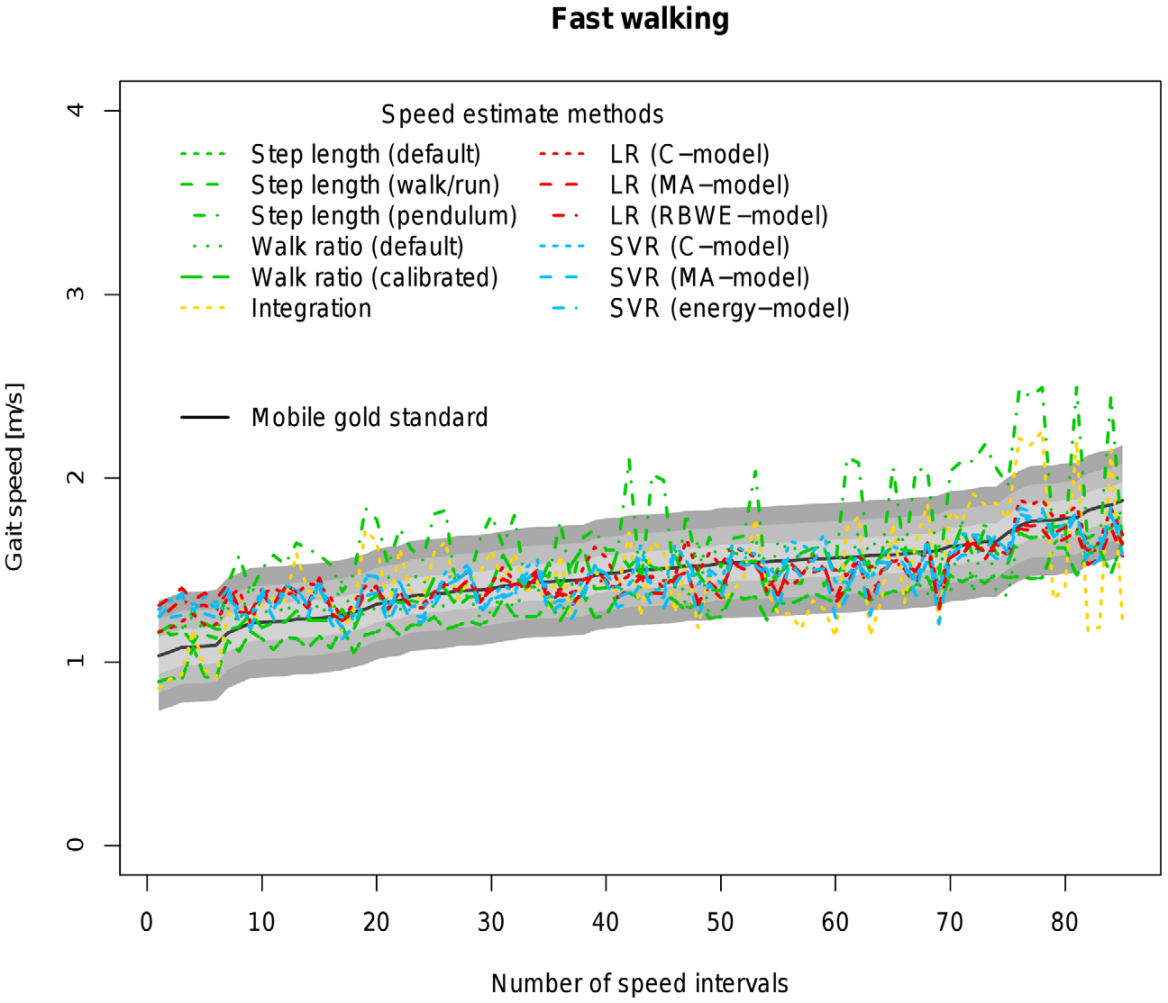
Visualization of coverage probability for fast walking in the indoor ecological validation. The black solid line represents speed as measured by the mobile gold standard for fast walking. Green, yellow, red and blue lines in different linestyles represent different speed estimates by different algorithms and models. The filled areas colored from light to dark grey around the black solid line indicate coverage probality levels from 0.1 to 0.3 m/s. Speed intervals are sorted increasingly across all participants for reasons of clarity and readability.

**Figure 5:**
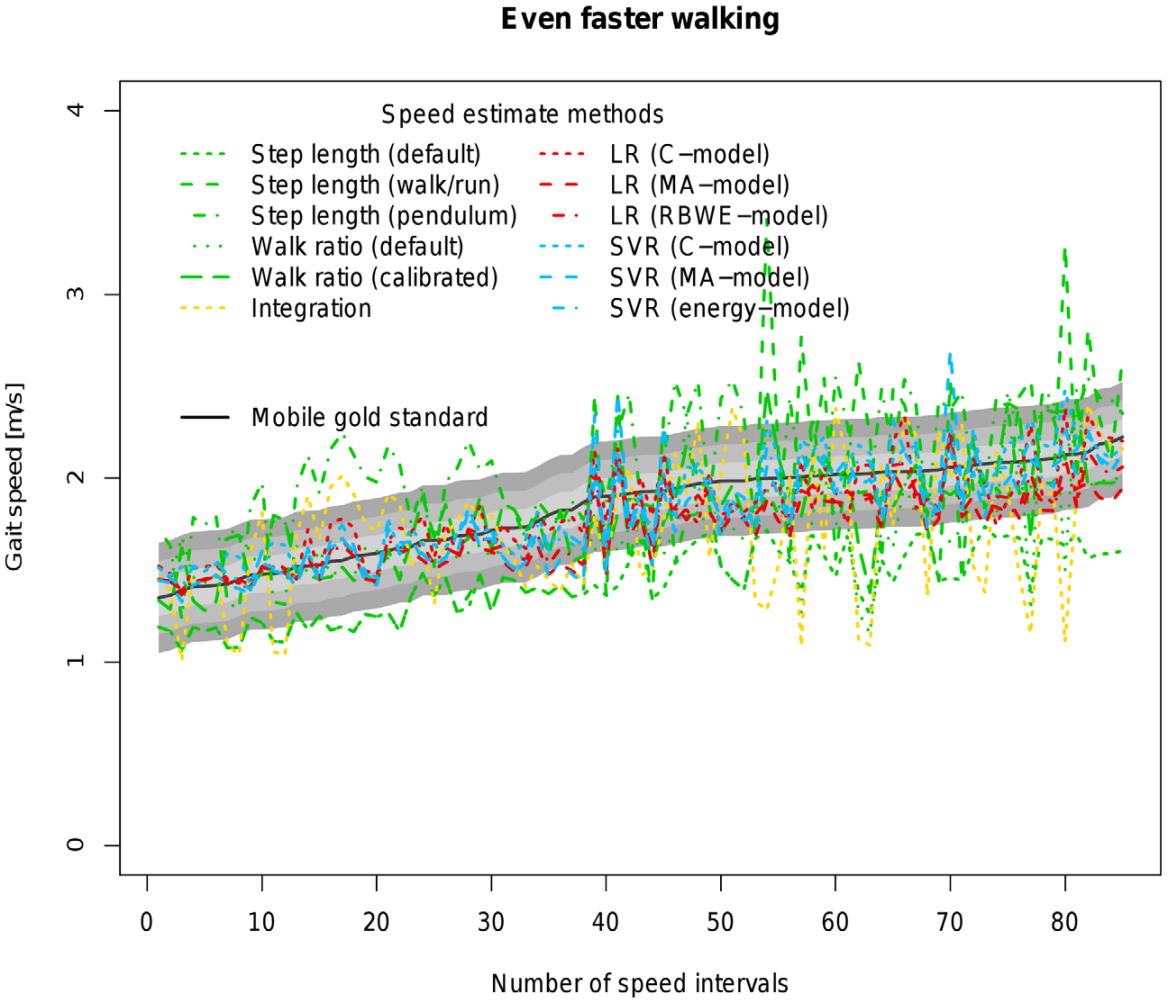
Visualization of coverage probability for even faster walking in the indoor ecological validation. The black solid line represents speed as measured by the mobile gold standard for even faster walking. Green, yellow, red and blue lines in different linestyles represent different speed estimates by different algorithms and models. The filled areas colored from light to dark grey around the black solid line indicate coverage probality levels from 0.1 to 0.3 m/s. Speed intervals are sorted increasingly across all participants for reasons of clarity and readability.

**Figure 6:**
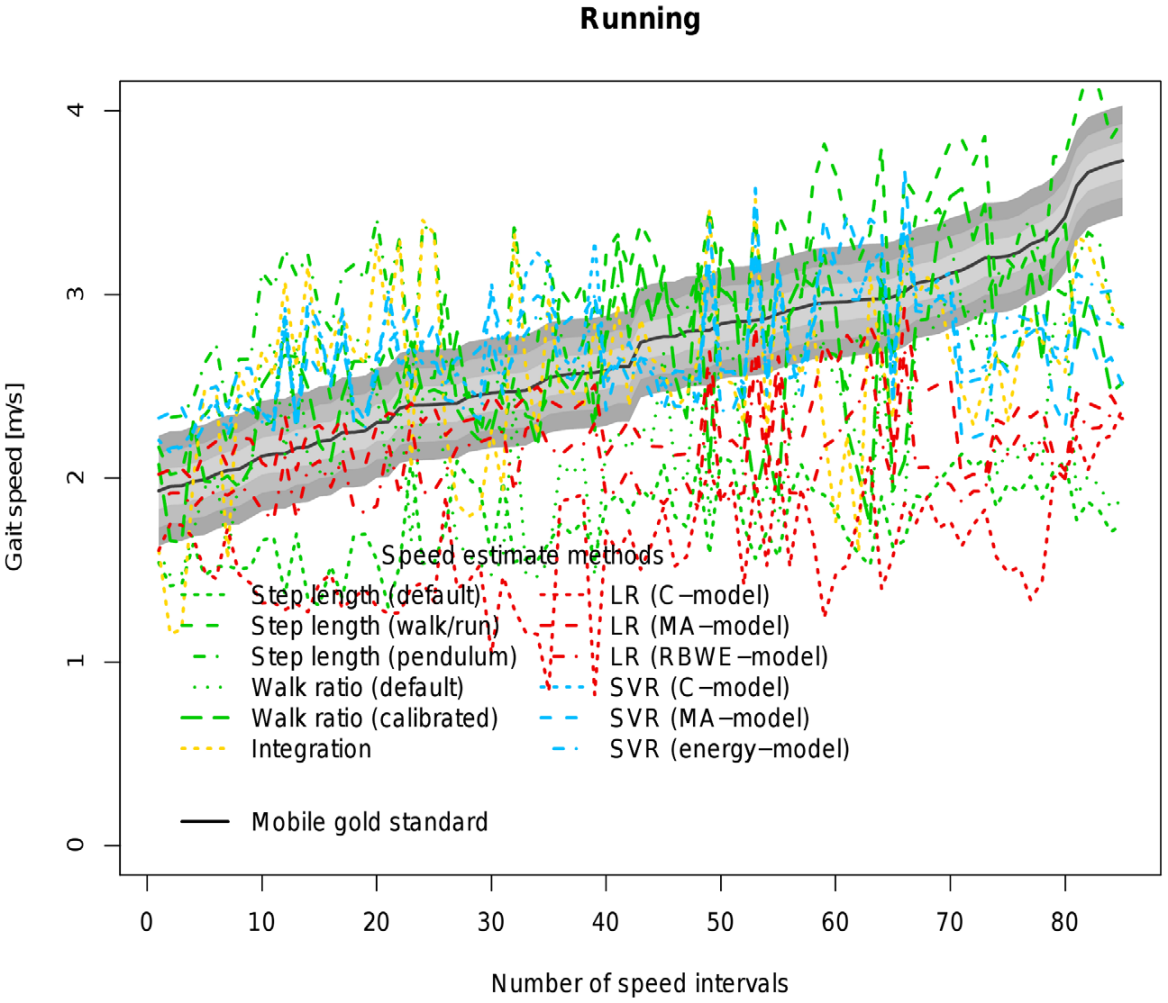
Visualization of coverage probability for running in the indoor ecological validation. The black solid line represents speed as measured by the mobile gold standard for running. Green, yellow, red and blue lines in different linestyles represent different speed estimates by different algorithms and models. The filled areas colored from light to dark grey around the black solid line indicate coverage probality levels from 0.1 to 0.3 m/s. Speed intervals are sorted increasingly across all participants for reasons of clarity and readability.

Table 4 allows a direct comparison of all available models based on coverage probability with tresholds ranging from 0.1 to 0.3 m/s as well as the concordance correlation coefficient between the estimated velocity of the respective algorithm and the velocity measured by the mobile gold standard. Figures 2, 3, 4, 5 and 6 visualize each algorithm's coverage probability for the five distinct speed levels.

**Table 4:** Algorithm ranking (indoor ecological validation including running).

Algorithm	CP1 (95% CI)	CP2 (95% CI)	CP3 (95% CI)	CCC (95% CI)
SVR (energy-model)	0.35 (0.10-0.58)	0.64 (0.35-0.89)	0.83 (0.52-0.98)	0.95 (0.94-0.96)
Walk ratio (calibrated)	0.33 (0.16-0.83)	0.60 (0.31-0.99)	0.79 (0.46-1.00)	0.95 (0.94-0.96)
SVR (C-model)	0.31 (0.07-0.52)	0.57 (0.24-0.84)	0.76 (0.41-0.97)	0.94 (0.92-0.95)
Step length (walk/run)	0.27 (0.17-0.44)	0.51 (0.33-0.76)	0.70 (0.48-0.92)	0.93 (0.92-0.94)
SVR (MA-model)	0.25 (0.11-0.41)	0.47 (0.26-0.72)	0.66 (0.41-0.89)	0.90 (0.88-0.92)
Integration	0.22 (0.03-0.54)	0.43 (0.09-0.88)	0.61 (0.17-0.98)	0.89 (0.87-0.91)
LR (RBWE-model)	0.21 (0.12-0.49)	0.40 (0.24-0.81)	0.57 (0.35-0.97)	0.81 (0.79-0.83)
Walk ratio (default)	0.21 (0.10-0.79)	0.41 (0.21-0.98)	0.58 (0.32-1.00)	0.85 (0.83-0.87)
LR (MA-model)	0.21 (0.12-0.34)	0.41 (0.24-0.63)	0.58 (0.35-0.83)	0.82 (0.80-0.84)
Step length (pendulum)	0.16 (0.01-0.29)	0.32 (0.04-0.54)	0.47 (0.16-0.74)	0.82 (0.79-0.84)
Step length (default)	0.15 (0.09-0.26)	0.30 (0.19-0.50)	0.43 (0.28-0.68)	0.65 (0.62-0.68)
LR (C-model)	0.14 (0.09-0.25)	0.27 (0.19-0.47)	0.40 (0.27-0.65)	0.53 (0.49-0.57)

Individual coverage probability with a maximum difference of 0.1 m/s (CP1) to 0.3 m/s (CP3) as well as concordance correlation coefficient (CCC) including 95% confidence intervals (95% CI) for each algorithm across all speed levels.

The non-linear support vector regression approaches outperform their linear counterparts (C-model, MA-model). Out of all SVR models, the energy-model performs best in terms of coverage probability which may be a hint that features for models should be selected according to bio-mechanical and sports science-based considerations rather than just “blind” automatic selection.

The top-performing SVR (energy-model) is closely followed by the walk ratio approach with individual calibration. However, it is evident (Figure 6) that running poses a problem to almost all algorithms indicated by a poor coverage probability which is to be expected from a biomechanical point of view. Furthermore running is not necessarily relevant in the context of our work with outcome parameters.

**Figure 7:**
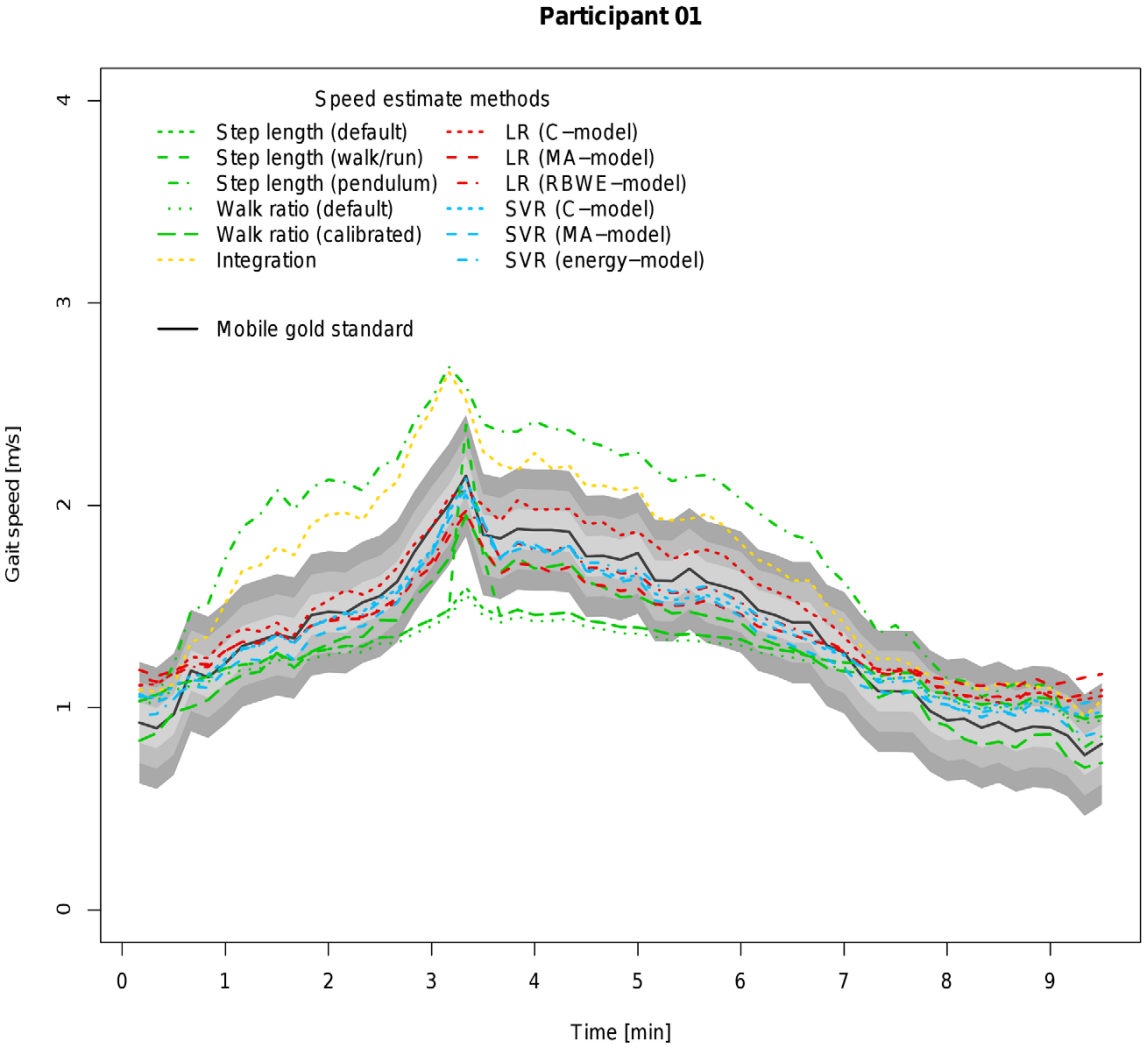
Visualization of coverage probability for participant 01 (male, 46 years) in the experiment for outdoor ecological validity. The black solid line represents speed as measured by the mobile gold standard for running. Green, yellow, red and blue lines in different linestyles represent different speed estimates by different algorithms and models. The filled areas colored from light to dark grey around the black solid line indicate coverage probality levels from 0.1 to 0.3 m/s.

**Figure 8:**
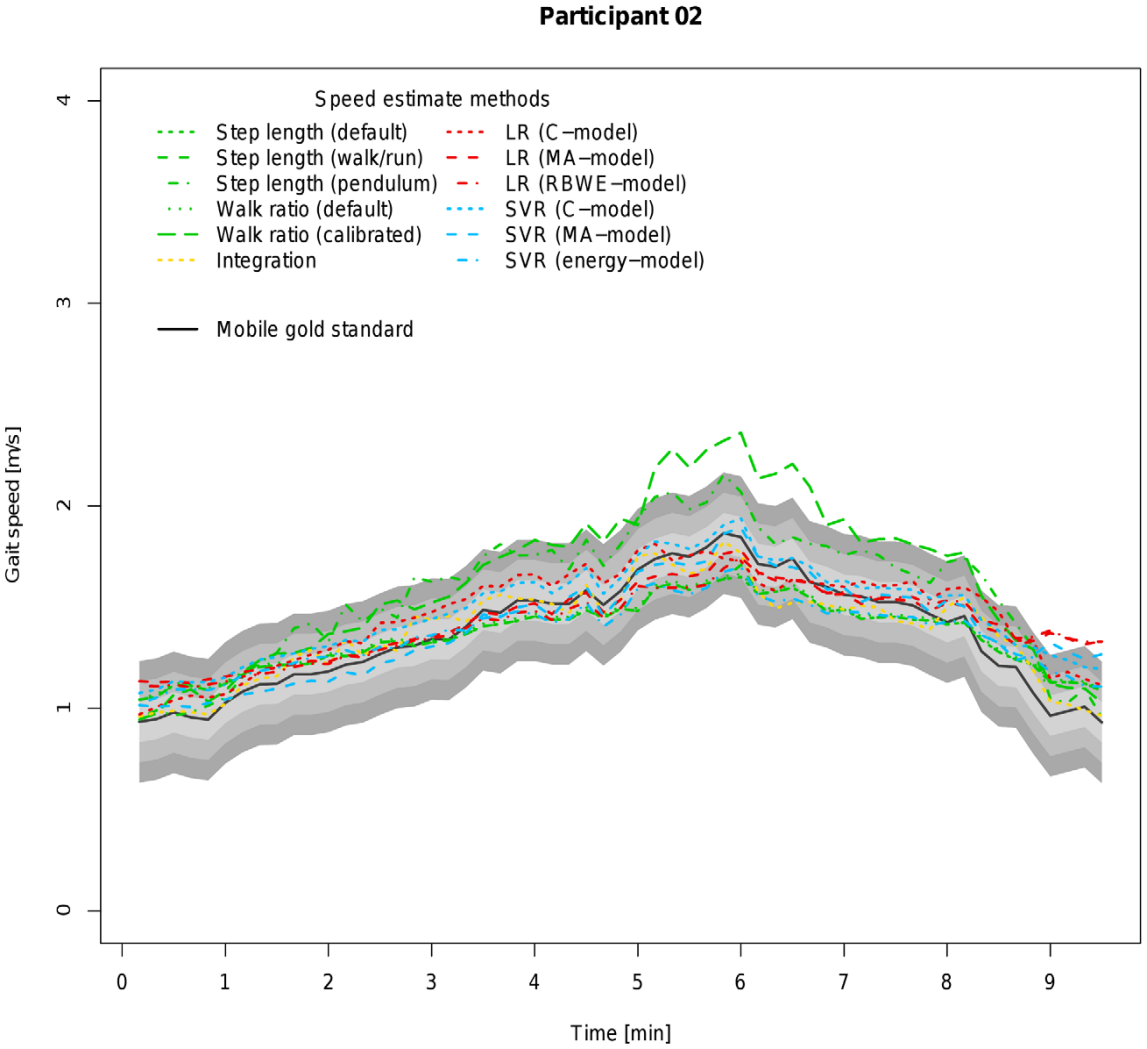
Visualization of coverage probability for participant 02 (female, 23 years) in the experiment for outdoor ecological validity. The black solid line represents speed as measured by the mobile gold standard for running. Green, yellow, red and blue lines in different linestyles represent different speed estimates by different algorithms and models. The filled areas colored from light to dark grey around the black solid line indicate coverage probality levels from 0.1 to 0.3 m/s.

For this reason, all parameters listed in Table 4 were re-evaluated after excluding the subsample of the indoor ecological validation involving running (Table 5). After exclusion of running, the calibrated walk ratio overtakes the SVR (energy-model). Considering that all other models do not require an individual calibration prior to their application and that one of the measurements (normal walking) of the indoor ecological validation experiment was used for calibrating step length and step frequency, it is not surprising that this unfairly boosts this algorithm's performance and introduces a bias in this method's favour. Looking at Table 6 and Figures 7 and 8 which display the results for the experiment to confirm the ecological validity outdoors, one can see that under conditions which approximate real-life situations SVR (energy-model) again outperforms all other algorithms, including the calibrated walk ratio approach. Further drawbacks of the calibrated walk ratio approach are the time and effort needed to calibrate the algorithm and the open question about how often this calibration process should be repeated in order to remain valid.

**Table 5:** Algorithm ranking (indoor ecological validation excluding running).

Algorithm	CP1 (95% CI)	CP2 (95% CI)	CP3 (95% CI)	CCC (95% CI)
Walk ratio (calibrated)	0.62 (0.36–0.89)	0.92 (0.65–1.00)	0.99 (0.84–1.00)	0.97 (0.97–0.98)
SVR (energy-model)	0.46 (0.12–0.70)	0.78 (0.48–0.96)	0.94 (0.73–1.00)	0.93 (0.92–0.94)
Walk ratio (default)	0.45 (0.12–0.92)	0.76 (0.41–1.00)	0.92 (0.62–1.00)	0.94 (0.92–0.95)
LR (RBWE-model)	0.39 (0.18–0.63)	0.69 (0.44–0.92)	0.87 (0.65–0.99)	0.89 (0.88–0.91)
SVR (C-model)	0.38 (0.06–0.69)	0.68 (0.27–0.95)	0.87 (0.59–1.00)	0.90 (0.88–0.92)
SVR (MA-model)	0.31 (0.14–0.59)	0.58 (0.32–0.86)	0.78 (0.50–0.95)	0.84 (0.81–0.86)
LR (C-model)	0.30 (0.15–0.61)	0.56 (0.33–0.91)	0.76 (0.51–0.99)	0.82 (0.79–0.84)
Step length (default)	0.30 (0.20–0.45)	0.56 (0.40–0.77)	0.75 (0.56–0.93)	0.79 (0.77–0.81)
Step length (walk/run)	0.30 (0.19–0.49)	0.57 (0.37–0.81)	0.76 (0.53–0.95)	0.85 (0.82–0.88)
Integration	0.29 (0.03–0.62)	0.54 (0.12–0.94)	0.73 (0.21–1.00)	0.85 (0.82–0.88)
LR (MA-model)	0.26 (0.13–0.57)	0.50 (0.28–0.83)	0.69 (0.43–0.93)	0.72 (0.69–0.75)
Step length (pendulum)	0.15 (0.00–0.28)	0.31 (0.02–0.52)	0.46 (0.11–0.72)	0.64 (0.59–0.69)

Individual coverage probability with a maximum difference of 0.1 m/s (CP1) to 0.3 m/s (CP3) as well as concordance correlation coefficient (CCC) including 95% confidence intervals (95% CI) for each algorithm across all speed levels excluding running.

**Table 6:** Algorithm ranking (outdoor ecological validity).

Algorithm	CP1	CP2	CP3	CCC
SVR (energy-model)	0.73	0.97	1.00	0.95
SVR (MA-model)	0.68	0.95	1.00	0.94
SVR (C-model)	0.64	0.94	1.00	0.94
LR (RBWE-model)	0.56	0.88	0.98	0.89
LR (C-model)	0.50	0.91	1.00	0.92
LR (MA-model)	0.50	0.82	0.96	0.85
Step length (walk/run)	0.41	0.72	0.90	0.78
Step length (default)	0.39	0.70	0.88	0.74
Walk ratio (default)	0.37	0.67	0.86	0.72
Walk ratio (calibrated)	0.34	0.62	0.81	0.81
Integration	0.30	0.58	0.78	0.81
Step length (pendulum)	0.12	0.28	0.48	0.68

Individual coverage probability with a maximum difference of 0.1 m/s (CP1) to 0.3 m/s (CP3) as well as concordance correlation coefficient (CCC) for each algorithm across all speed levels.

In Table 5 SVR (energy-model) is closely followed by the default walk ratio approach which is a computationally much simpler method and would therefore be more favourable for real-time data processing embedded in the device's firmware. However, the outdoor ecological validity for computationally simpler methods including walk ratio or step length approaches fails (Table 6), stabilizing SVR (energy-model) 's position on top of the ranking list.

A potential drawback of the newly developed mobile gold standard is the fact that the device may constrain the natural arm swing inherent to human gait. However, in experiments conducted by the authors using a treadmill at a fixed speed with and without perambulator, evidence suggests that the centre of mass at which the actibelt® is placed is only marginally (if at all) affected and that therefore the difference between the two conditions is negligible. Another limitation of the mobile gold standard is the so-called boundary effect. Due to the speedometer which is dependent on the wheel's (full) revolution, these boundary effects can occur while starting or stopping, i.e. measurements in the first/last few seconds may not be fully accurate. For this reason, only the middle portion of the acquired data was used for analysis.

A novel method for measuring self-selected walking speed using support vector regression combined with mobile accelerometry data using actibelt® technology was developed and validated. Accuracy and precision are high with a CCC of 0.93 (95%CI 0.92–0.94) and a CP1 of 0.46 (95%CI 0.12-0.70) for a deviation of 0.1 m/s when compared to the mobile gold standard while walking indoors. This new method allows the assessment of self-selected walking speed in free-living individuals over a prolonged period of time which may increase confidence in the use of walking speed as a patient-oriented outcome measure.

Only recently, the construct validaty of our new method has been shown in a study involving healthy blood donors [40]. Further steps towards demonstrating that walking speed can be a new outcome and effective measure require comparisons of walking speed measured in short distance walk tests and long-term measurements as well as an extension of experiments to confirm the ecological validity in free-living conditions for diseased subjects. For this purpose, future studies will need to determine the ability of walking speed to discriminate between different levels of disease progression and assess the accuracy of walking speed provided by the actibelt® under controlled conditions such as the 6-minute walking test across levels of walking ability. We plan to perform studies with multiple sclerosis patients with different levels of disability status as a representative for other chronic disabling diseases as a precursor to the method's application administered under real free-living conditions. Longitudinal studies could provide pivotal information about test-retest reliability of the new method of walking speed assessment, the ability of walking speed to detect changes of disease status and the potential use as surrogate variable.
